# morphforge: a toolbox for simulating small networks of biologically detailed neurons in Python

**DOI:** 10.3389/fninf.2013.00047

**Published:** 2014-01-28

**Authors:** Michael J. Hull, David J. Willshaw

**Affiliations:** Institute for Adaptive and Neural Computation, School of Informatics, University of EdinburghEdinburgh, UK

**Keywords:** multicompartmental modeling, biophysical modeling, small neuronal network, code-generation, Python, toolbox

## Abstract

The broad structure of a modeling study can often be explained over a cup of coffee, but converting this high-level conceptual idea into graphs of the final simulation results may require many weeks of sitting at a computer. Although models themselves can be complex, often many mental resources are wasted working around complexities of the software ecosystem such as fighting to manage files, interfacing between tools and data formats, finding mistakes in code or working out the units of variables. morphforge is a high-level, Python toolbox for building and managing simulations of small populations of multicompartmental biophysical model neurons. An entire *in silico* experiment, including the definition of neuronal morphologies, channel descriptions, stimuli, visualization and analysis of results can be written within a single short Python script using high-level objects. Multiple independent simulations can be created and run from a single script, allowing parameter spaces to be investigated. Consideration has been given to the reuse of both algorithmic and parameterizable components to allow both specific and stochastic parameter variations. Some other features of the toolbox include: the automatic generation of human-readable documentation (e.g., PDF files) about a simulation; the transparent handling of different biophysical units; a novel mechanism for plotting simulation results based on a system of tags; and an architecture that supports both the use of established formats for defining channels and synapses (e.g., MODL files), and the possibility to support other libraries and standards easily. We hope that this toolbox will allow scientists to quickly build simulations of multicompartmental model neurons for research and serve as a platform for further tool development.

## 1. Introduction

### 1.1. Why build biophysical models?

Numbers are central to science and the scope of testable predictions generated from quantitative theories is much broader than from qualitative ones. The building of computational models of simple nervous system preparations in conjunction with experimental work has a history of uncovering principles which are germane across all of neuroscience, for example: action potential initiation and propagation (Hodgkin and Huxley, 1952; Moore et al., 1983; Faisal and Laughlin, 2007); the effect of ion channels types and distributions on the excitability of neurons (Dodge and Cooley, 1973; Hurwitz et al., 2008; Hudson and Prinz, 2010); the neuronal basis of sensory pathway integration (Baxter et al., 1999; Cataldo et al., 2005); and the intrinsic maintenance of rhythmic activity by central pattern generators (Perkel and Mulloney, 1974; Getting, 1983; Calin-Jageman et al., 2007; Roberts et al., 2010; Doloc-Mihu and Calabrese, 2011).

Unfortunately at the level of individual neurons and synapses, in general it has been difficult to experimentally reverse engineer neuronal networks and recreate realistic behavior reliably in computer simulations. The functions of these systems do not seem to partition neatly into modules, often contain redundancy, and in many cases it is impossible to perform the ideal experiments. Biological systems contain many complex interactions, are highly non-linear, and are governed by large numbers of parameters, many of which are difficult to determine experimentally. Although modeling can lead to conceptual insights (Hillis, 1993), arguably what is useful in modeling studies is not a single, final, polished model, but instead the results that emerge during its construction. The process of modelling can highlight ambiguities in experimental data and the robustness of a particular phenomenon in a model can also give insight into how tightly tuned the parameters of a system need to be (Marder et al., 2007). Moreover, modeling allows investigation of phenomena that cannot be explained by existing hypotheses and *in silico* experiments provide a way to ask questions that cannot be addressed *in vivo* to test new hypotheses.

One way to test how well a computational model reflects reality is to perform many experiments *in vivo* and *in silico* and compare their results. One well documented approach to this is to construct databases containing the results of high dimensional parameter sweeps, which allows regions of parameter space to be investigated (Prinz et al., 2003; Doloc-Mihu and Calabrese, 2011). Another approach is the use of optimization algorithms in conjunction with simulators to automate the search for parameters underlying model neurons whose behaviors match those observed experimentally (Geit et al., 2008). In other situations, a more interactive workflow may be more appropriate. The parameter spaces might be simply too large or the simulations too computationally intensive to allow the space to be mapped out by brute force. The modeler may also want to adjust parameters manually, either to get an intuition about the robustness of a set of parameters or to perform specific *in silico* experiments on individual components. For networks that have been well characterized electrophysiologically, the number of experiments that could be replicated may be quite large. One such example is the neuronal network that drives swimming in the hatchling tadpole [reviewed in Roberts et al. 2010]. The pattern of motor-neuron activity in the animal during fictive swimming is well defined and is therefore one constraint on any simulated model network, but the effects of other experimental protocols such as the responses of isolated synaptic and channel currents to voltage clamps, the responses of individual neurons to step current injections, the coupling coefficients and frequency responses of paired recordings of electrically coupled neurons, the effects of chemical synapse and gap junction agonists and antagonists on neuronal and network behavior, and the effects of hemisection and other lesioning experiments are experimentally characterized and can be replicated in modeling.

### 1.2. Existing neuronal simulators

**Figure 1:**
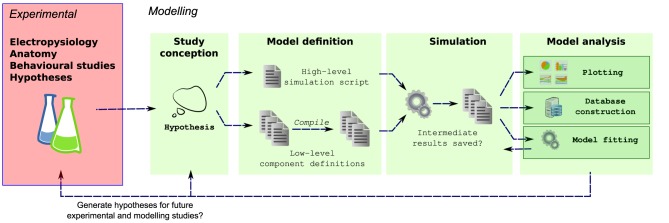
**Multiple steps using a variety of libraries, tools and languages are often required from the conception to the evaluation of a modeling hypothesis**. During *model definition*, the computational model must be defined for a specific simulator (e.g., NEURON), which can involve writing several interdependent files in different, domain specific languages, and possibly compilation of these files for simulation efficiency. Next, the simulator is invoked, which may write results to files on a disk. These results are then further analyzed, for example by plotting of comparison traces against physiological records, constructing of databases or automatically fitting model parameters.

Efficient simulators already exist for simulating populations of multicompartmental neurons, for example NEURON (Carnevale and Hines, 2006), GENESIS (Bower and Beeman, 1998) and MOOSE (Ray et al., 2008). These simulators are highly optimized for solving neuronal models and allow the behaviors of a wide range of networks to be investigated. Simulation of complex neuronal networks is computationally intensive because it requires solving large numbers of differential equations. For simulation speed, these equations should be written in a language that can be compiled to efficient machine code, and therefore many simulators split the definition of a simulation into two parts: an interpreted language for defining the overall simulation setup and compilable language for defining the equations governing neuron and synapse models. [e.g., NEURON: HOC/MODL; NEST: PyNest, C++; PCSIM (Carnevale and Hines, 2006; Eppler et al., 2008; Hines et al., 2009; Pecevski et al., 2009)]. This approach lets modelers quickly build simulations in high-level languages while allowing the simulator kernel to solve equations efficiently. Unfortunately this also fragments model definitions into different files and languages which can also make simulations harder to maintain (Figure 1).

### 1.3. Managing complexity

The issues raised in managing a large collection of similar simulations go beyond solving sets of equations. These systems are complex and contain large numbers of both parameters and permutations of possible scenarios. To address scientific questions by developing and testing computational models, what is needed are tools that make it simple to build and manage components and simulations that can be quickly adapted to perform specific experiments. The time it takes for simulations to execute is an obvious bottleneck on a modeler's productivity, but other important surrounding issues can also drain their time, for example: tracking the providence and further development of models, sharing models with colleagues, connecting simulation tools together, managing intermediate data and files and being able to reliably and quickly reproduce results. Moreover, in the modeling use-cases outlined above, the same, or similar components are used in many simulations, meaning the definitions of these components must either be replicated, or shared between simulations. When these issues are taken together, managing the data dependencies between large numbers files can quickly become overwhelming (Hudson et al., 2011). It is important to acknowledge these softer issues and overcome them in order to allow effective, sustainable model development. An experimentalist requires suitable instruments to effectively address scientific questions - similarly a modeler requires suitable tools to effectively build and manage biologically realistic models and variations. Specifically, we need to remove user intervention from mundane activities, and develop tools and toolchains to manage simulations and data which support reproducible workflows and encourage the reuse of model components and algorithms.

The need to remove unnecessary complexity to keep the scientific questions the focus of attention (Brette, 2012) has been recognized by the neuroinformatics community. Different approaches are used to simplify the interfaces to existing simulators which offer different trade-offs in the ease of reusability of components, the complexity of the surrounding toolchains, and in the expressiveness and readability of the model specifications. One approach has been to design high-level, declarative languages for expressing the mathematical behaviors of neurons and synapses, for example MODL (Carnevale and Hines, 2006), NDF (Bower and Beeman, 1998), NeuroML/LEMS (Goddard et al., 2001; Gleeson et al., 2010) and NineML (Raikov et al., 2011). These languages are either read natively by the simulator, or intermediate code-generation tools can be used to translate these descriptions to run on existing simulators. Another approach has been to develop high-level programmable interfaces to existing tools. One example is PyNN (Davison et al., 2008), a Python object-model which allows simulations of networks of single-compartment neurons to be defined and run on multiple simulators (e.g., NEURON, NEST, MOOSE). A more interactive approach was taken by neuroConstruct (Gleeson et al., 2007), which allows simulations of networks of both single and multicompartmental neurons to be defined from using a graphical interface tool written in Java and can generate appropriate scripts to run on simulators including NEURON, GENESIS, MOOSE and PSICS (Cannon et al., 2010).

### 1.4. The goals of morphforge

morphforge is a high-level Python library for building simulations of small populations of multicompartmental neurons, in which membrane voltage is calculated from the sum of individual ionic currents flowing across a membrane. The focus of the library is to make it easy to construct and maintain simulations of small populations of neurons and synaptic connections, with particular focus on: (a) allowing simulation specification, with visualisation and analysis in a minimal, clean, human readable language; (b) reducing complex simulation toolchains to a single Python script; (c) promoting reproducible research through automatic documentation generation from models; (d) facilitating parameter sweeps by allowing multiple independent simulations to run in a single script; (e) encouraging the reuse of components such as morphologies, neurons and channels so that specific and stochastic variation in parameters is simple; (f) transparent handling of different units; (g) allowing the use of established formats, (e.g., MODL files), but also simplifying the definition and sharing of new channel types, including the possibility of supporting existing libraries and standards easily. morphforge is simulator-independent in the sense that the abstract concepts are well separated from the NEURON implementation in the codebase, which would make it simple to create backends for other simulators (e.g., GENESIS), generate compilable C-code, or to map simulations to more esoteric hardware platforms such as GPGPUs or SpinNaker (Painkras et al., 2012).

morphforge is not a simulator itself; it is a high-level interface to simulators (currently NEURON) and provides a set of high-level primitives for specifying morphologies, channel distributions and network connections in Python. morphforge is not designed for large-scale simulations and a design choice was taken to prioritise the interface to the modeler over simulation speed. morphforge provides a single interface for building models of multicompartmental neurons: an entire *in silico* experiment, including the definition of neuronal morphologies, channel descriptions, stimuli and plotting of results can be written in a single short Python script. Reusability and explicit component variation was a central concern and consideration has been given to the reuse of both algorithmic and parameterizable components. The design of the object-model allows models to be written in a declarative style, and because a Python object-model is exposed, tools can be built on top of morphforge. Existing simulators support a wide range of features and morphforge does not try to incorporate them all into its own interface. However, care has been taken to ensure that these features are still accessible, for example it is possible to insert custom HOC commands or MODL files directly when using NEURON as a backend. Instructions for installing morphforge can be found at http://morphforge.readthedocs.org.

## 2. Example: current injection into a passive cell

**Figure 2:**
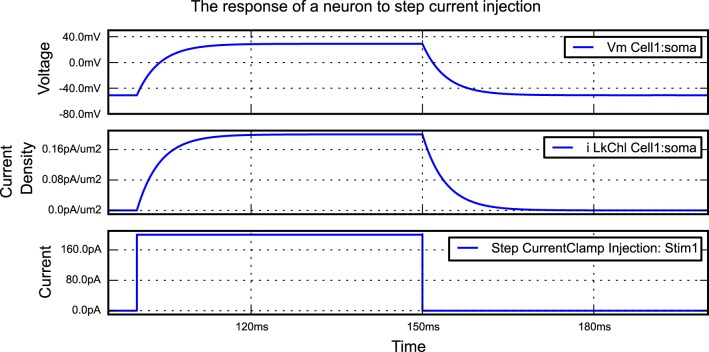
**The figure produced by the code in Listing 1**. The three recorded variables are automatically plotted on separate axes with corresponding units.

A simple morphforge script is shown in Listing 1 which simulates the injection of step current into a single compartment neuron with leak channels. When this script is run, it generates the graphs shown in Figure 2. First, it is specified that NEURON will be used as a backend, and a Simulation object is created which will last for 200 ms. Next, a Cell object is created. The morphology is defined using a built-in helper function which produces a single Section (discussed below) of 1000μm^2^, although this morphology could also come from another source such as an SWC file or built programmatically in Python. A leak Channel object is defined with a conductance and reversal potential (line 13). This leak channel is applied to the surface of the neuron, and the capacitance of the neuron is also set. A step current-clamp is created and inserted into the neuron at the soma. It is specified that the membrane voltage, the current-density of the leak channel at the soma, and the current injected by the current-clamp should be recorded. At this point (line 28), an object model of the simulation has been created in memory. When run is called, since the NEURON backend is being used, morphforge carries out a series of actions behind the scenes. A HOC file for the simulation is created and a new process is launched. The relevant MODL files for the leak channel are generated, compiled and registered with the new process, and NEURON runs. The relevant data is saved during the simulation, and is returned to the calling process as numpy^1^ arrays within a SimulationResults object (i.e., results). Finally, all plotting is performed automatically by the TagViewer class. This class inspects the SimulationResults objects and, by default, plots different Traces on different axes depending on their type (discussed below), including appropriate units.

**Listing 1:**
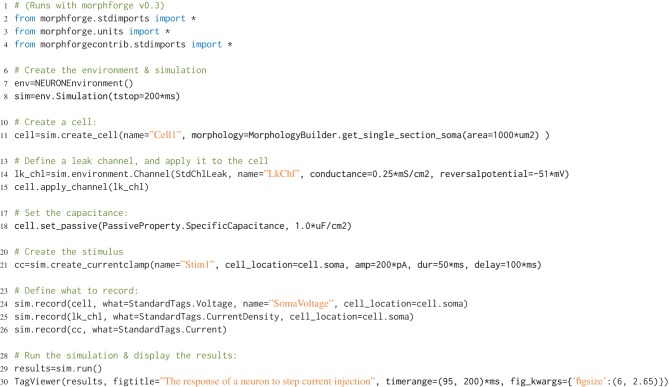
**An example of a simple simulation in morphforge**. A single compartment neuron with leak channels is created and a step current injected into it. The membrane voltage, current density and conductance density are recorded. The resulting graphs generated by this example are shown in Figure 2.

## 3. The morphforge package

### 3.1. Architecture overview

Modern simulators such as NEURON support a range of features, from modeling the internal diffusion of ions within a multicompartmental neuron to the calculation of extracellular potentials. It would require a huge number of resources to implement a new interface that was the superset of these features. Rather than try to define a single monolithic system, the approach taken in morphforge is to provide a collection of classes and interfaces which form the core infrastructure, and then use a system of plugins which can be written to implement particular features. For example, morphforge is agnostic to how a synapse model is defined. The core of morphforge defines a minimal interface, and then plugins can be written, which allow a synapse specified in Python or MODL for example to be used with NEURON. This means morphforge naturally splits into two parts, *morphforge-core*, which contains the core infrastructure, and *morphforge-contrib*, which contains for example the plugins that define how a synapse model specified in a particular format is mapped to a particular simulator backend.

morphforge is split into four layers each defining a set of classes that work together as an object-model (Table 1). The higher layers depend on the lower levels, but lower levels do not need the higher ones, for example, Morphology objects are used by the *simulation*-*layer*, but can also be used without it, for example for anatomical reconstructions. The *core-layer* provides a single point of access to control random number seeding (for the script and simulations), simulation settings and locations on the filesystem access as well as the plugin infrastructure and utility functions. The *morphology*-*Layer* provides classes that represent neuronal morphologies as a tree of cylinders and functions for their creation, import, export, rendering, traversal and manipulation. The *simulation*-*layer* defines a high-level object-model on top of the morphology objects for defining multicompartmental neurons with complex channel distributions. Primitives for defining network connectivity and an interface for recording values during a simulation are also provided. It provides a set of component libraries to allow objects, such as morphologies, channels and synapses, to be defined once and reused with different parameters as well as an extensible high-level object-model for representing analog signals with units. Finally, the *simulationanalysis*-*layer* provides functions for analysing the output of simulations such as spike detection, a visualization system for easily viewing the outputs of simulations and infrastructure for automatically generating summaries of simulations including the details of components such as channels and synapses.

**Table 1:** **An overview of morphforge's architecture**.

	**mf-core**	**mf-contrib**
Simulation analysis	Plotting library Summary generation infrastructure	Trace analysis functions (e.g., spike counting)
Simulation	Classes for defining simulations (e.g., Cell, Synapse, GapJunction, Channel) and results (e.g., Trace, Event) NEURON backend support	Mapping of synapse and channel descriptions to simulators, e.g., NEUROML → NEURON
Morphology	Classes for defining neuronal morphologies (e.g., Morphology, Segment, Region, MorphLocation)	Import/export of morphologies (e.g., SWC) Visualization
Core	Random number seeding and generation File IO	

*morphforge is divided into two packages: core and contrib, and also into four layers: core, morphology, simulation and simulationanalysis. In general, the core package contains the infrastructure for the object model, while the specific functionality such loading from particular file formats or specific analysis functions are implemented in the contrib-package*.

The object-model underlying morphforge is designed to be flexible and extensible, but for conciseness, default parameters and syntactic sugar methods have been introduced for common scenarios. For example, morphforge uses a flexible model for defining stimuli and *morphforge-contrib* defines different current- and voltage-clamp protocols, including step, ramp and sinusoidal current injections. These current-clamps are all created in morphforge by calling the method Simulation.create_currentclamp(). By default, this method will create a step current-clamp, but other, possibly user-defined, protocols can be specified instead by supplying an appropriate protocol argument, as shown in Listing 2.

**Listing 2:**
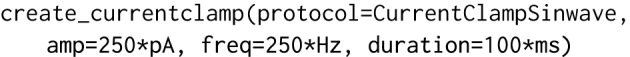
**Specifying a sinsusoidal current-clamp in morphforge**.

In the next sections we describe some features of morphforge which simplify the building and management of simulations by making intentions more explicit, facilitating reuse of components and removing mundane code from scripts. Specifically, these include: the reuse of model components using component libraries; the explicit use of units in parameters and results; a simple system for selecting results traces and a high-level plotting interface; generation of summary PDF documents of components and simulations; and a high-level notation for defining distributions of channels over the surface of neurons. Finally, the testing infrastructure for morphforge is briefly discussed and it is demonstrated how the object-model can be used as a basis to build more complex tools.

### 3.2. Interesting techniques used

#### 3.2.1. Component libraries

In order to produce maintainable software, information should be specified only once, so that any future changes only need to be made in a single place (McConnell, 2004). Similarly in modeling it is important to keep as few copies of a model as possible, so that if experimental work generates a revised estimate for a parameter, the model only needs to be changed in a single place. However, for modeling to be valuable, we often need to be able to produce variations on a basic model component in order to investigate the effects of parameters and simulate specific experiments. morphforge supports the reuse and variation of model components by providing four libraries: MorphologyLibrary, ChannelLibrary, PostSynapticReceptorLibrary and CellLibrary. An example is shown in Listing 3.

**Listing 3:**
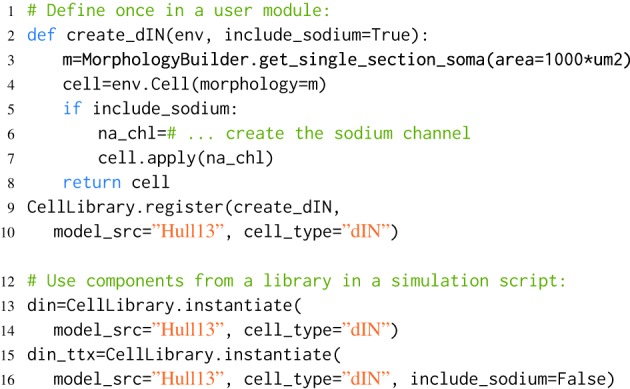
**An example of component libraries in morphforge**. First a function to create a particular cell type is defined (lines 2–8), and registered with the CellLibrary (lines 9,10). (Typically, this would be written once in an imported module). Next, cells can be created by referring to the model_src and cell_type in simulation scripts (lines 12–16). Since any arguments passed to instantiate are forwarded to the user-functions, this make it possible to produce variations of a Cell. An example is shown for the parameter include_sodium, to produce a model of a cell in the presence of Tetrodotoxin (TTX) (line 15-16).

When morphforge is initially imported into a script, these component libraries are empty. The modeler writes a function that builds a particular component (e.g., create_dIN (line 2)), and registers this *builder*-function with morphforge (line 9), including a reference to the model_src and the type. Next, in the simulation setup part of the script, it is then possible to explicitly build model components (line 12). The component libraries automatically forward additional keyword-arguments to the builder-functions which allows components to be easily parameterized, for example, Listing 3 shows how the optional parameter include_sodium could be used to instantiate variants of the neuron without sodium channels, without needing to copy-and-paste the entire builder-function.

Typically, the builder-function definition and registration is performed in a separate Python module written by the user, to allow the same component (or variations such as removing the sodium channels to simulate the effects of TTX as shown in Listing 3) to be referenced by name and used in multiple simulation scripts. Using explicit string references can make the intention of the simulation clearer. Component libraries also simplify the tracking of components that are in used and it is possible to iterate over all the registered components in a library (synapses, channels, morphologies) which can be summarized to a PDF or HTML file by calling summary_table(). Model databases such as ModelDB (Hines et al., 2004) and Channelpedia (Ranjan et al., 2011) are important resources for the modeling community, making it easier to reproduce and reuse simulations and components. Component libraries in morphforge provide a platform for individual modelers to organize subcomponents as isolated components in distributable Python packages which opens up further possibilities for component sharing in the community.

#### 3.2.2. Embedded units

Quantities in neuroscience are expressed in a variety of units; for example conductance densities can be specified in pS/μm^2^, mS/cm^2^ or even indirectly: “a neuron with surface area of 1200μm^2^ with an input resistance of 300 MΩ.” Although the conversions between these quantities are not complex, mistakes can be made when making these tedious conversions by hand. morphforge uses the quantities^2^ package to handle these conversions transparently. All input parameters and output Trace objects in a simulation have associated units, for example see Listing 1 and Figure 2. Moreover, morphforge provides high-level plotting primitives that transparently take care of plotting units on graphs (described below).

#### 3.2.3. Channel and synapse definitions

Several specialist file formats already exist for defining membrane channel and synaptic dynamics in simulators, for example MODL (Carnevale and Hines, 2006), NeuroML (Gleeson et al., 2010), NineML (Raikov et al., 2011), and in some cases it is important to be able to define channels and synapses in code directly (e.g., see graphical tool below). Rather than choosing a single format for specifying the dynamics of components, morphforge leverages Python's dynamic typing to support a flexible model for membrane channels and synapses which is agnostic about the underlying format.

In the case of membrane channels, it is assumed that an abstract *channel* has a set of parameters, which may vary in different areas of the membrane, and there is a default value for each of these parameters. To integrate with the morphforge framework, Channel objects are expected to provide a particular interface, some methods of which are general and some of which are simulator-backend specific. All Channel objects must implement the methods get_variables() and get_defaults() which return the list of associated parameter names, (for example: [“gbar”,“erev”]) and their default values respectively. These are used by the channel-distribution infrastructure in morphforge when calculating the parameter values which should be applied to each compartment of a Cell (described below). Additionally, when the NEURON backend is used, Channel objects must also implement the methods create_modfile() and build_hoc_section(), which build the MODL code and insert the relevant code into the HOC file. A similar approach is taken for synapse models.

One advantage of this abstraction is that it allows channels specified in different formats, for example NeuroML and MODL, to be used within a single simulation, as shown in Listing 4. This provides an incremental pathway for the translation of models from one format to another.

**Listing 4:**
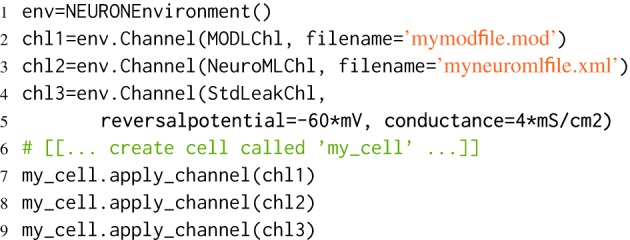
**Using different types of channel within a single simulation**. MODLChl, NeuroMLChl, StdLeakChl are Channel classes, defined in *morphforge-contrib*. which allow components specified in MODL, NeuroML and as explicit parameters, to be used with the NEURON backend.

#### 3.2.4. Channel distribution notation

In many neurons it is known that the distribution density of a particular type of channel over the membrane is not uniform. morphforge allows us to specify that specific types of Channels are distributed with different parameters over specific neuronal regions. For example, the conductance density of potassium channels might be 30 mS/cm^2^, except in the apical dendrites where it is 50 mS/cm^2^. Existing models have used even more complex channel distribution schemes, for example where the density of sodium channels on the initial segment of the axon varies as the function of distance from the soma (Schmidt-Hieber et al., 2008).

morphforge uses similar terminology to NEURON in reference to morphologies: briefly a Section is an unbranched region of membrane approximated as a conical frustrum that can be further divided into *segments* to increase simulation accuracy (Carnevale and Hines 2006; segmentation in morphforge is described in the documentation). morphforge uses a high-level notation to allow complex specifications of channel densities over neurons. This is achieved by passing a triplet of objects: (Channel, Applicator, Targeter), to the apply_channel method of Cell objects. The Targeter object defines which Sections in the Cell this triplet applies to (i.e., a predicate object). The Applicator object defines how the parameters of the Channel should vary over the specified Sections. Listing 5 shows an example in which twice the density of potassium channels are applied in the “dendrites” as in the rest of the neuron. In this example, we use two Targeters: TargetEverywhere and TargetRegion, and one Applicator: ApplyUniform. A Channel object has an associated set of default parameters (e.g., gbar), which are used by default by ApplyUniform (e.g., line 4), although they can be overridden or scaled (e.g., line 9). The apply_channel method can be called many times for the same Channel on the same Cell, with different Targeters and Applicators. However, in the simulation, a particular Channel will only be applied once to any given Section. When multiple Targeters affect the same Section, morphforge uses a system of priorities to determine which triplet to use. By default, triplets containing Targeters which are more area specific have a higher priority. Therefore in Listing 4, the triplet containing TargetRegion overrides that containing TargetEverywhere. This scheme will also allow a channel to be distributed with a continuously varying parameter across a neuronal membrane (i.e., different for each segment of a Section), for example, in order to distribute a type of sodium channel along the initial part of the axon in which the channel density is specified as a function of distance from the soma, although this has not yet been implemented.

**Listing 5:**
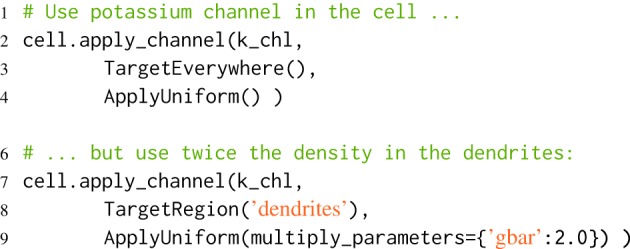
**Defining distributions of channels over neuronal morphologies**. First, it is specified that k_chl-Channels should be applied uniformly over all the neuron (line 2), then it is specified that they should also be applied to the dendrites with twice the default value of gbar. Since TargetRegion has a higher priority than TargetEverywhere, the second triplet is used to determine the value of gbar in the dendrites.

This notation offers advantages over manually defining the densities for each Section, traditionally achieved using for loops and if statements. Firstly, this means that a channel can be switched off by editing a single line. Secondly, when summary documents of simulations are created (below), this notation is simpler to interpret compared to explicit lists of parameter values per Section, which may be very long.

#### 3.2.5. Trace selection using a system of “tags”

In a simulation of a small network, the modeler may want to visualize the internal states of many neurons and synapses–how do we effectively specify which values to plot or use in other forms of analysis? In many simulators, the variables that should be recorded are specified during the simulation setup phase and the results can then be retrieved after the simulation has run for further processing. This approach is also taken in morphforge. During simulation setup, the modeler specifies which values to record(), and this will cause the corresponding time-varying analog waveforms, which are encapsulated in Trace objects, to be returned after the simulation has run. morphforge introduces a system of tag-selection strings in order to quickly find particular Trace objects that were recorded during a simulation. To facilitate this, each Trace object contains a set of strings called *tags*, which contains contextual information about it. These tags can be specified explicitly by the user in the call to Simulation.record(), and morphforge will also add certain tags automatically, for example the tags *Voltage* or *CurrentDensity* will be added if the Trace object represents a voltage or current density recording. After the simulation has run, tag-selection strings can be used to select specific sets of Trace objects. The tag-selection string uses a simple language with the keywords: ALL, ANY, AND, OR and NOT. The terms ALL{A,B,..,C} and ANY{X,Y,...,Z} are matching predicates which take arguments separated by commas. ALL{A,B,..,C} returns whether a particular Trace contains all the tags specified (i.e., A, B and C) and ANY{X,Y,...,Z} returns whether a Trace contains any of the tags specified (i.e., X, Y or Z). These match predicates can be joined with the AND, OR and NOT operators as well as brackets to allow more complex queries. For example, ALL{Voltage} will return all the voltages recorded in the simulation and ALL{CONDUCTANCE,SYNAPTIC,PRE:cell1,POST:cell 2} AND ANY{NMDA,AMPA} can be used to retrieve all Trace objects representing conductances in AMPAR and NMDAR synapses from *cell1* to *cell2*. This system of tagging, and the use of conventions (such as voltage traces always have a *Voltage* tag) allows looser coupling between different parts of the code and allows scripts to be more concise.

#### 3.2.6. Automatic plotting of results

The Trace objects contain methods which can return numpy arrays of their times and data for analysis and plotting using other scientific Python libraries. morphforge also provides a class, TagViewer, which makes plotting a selection of Trace objects from a simulation more succinct. The output of the TagViewer is a single figure, containing a series of axes each with the same time base. An example is given in Listing 6. The details of each axis, such as which Traces should be plotted, the y-label, the appropriate display range and unit are specified by PlotSpec objects (see Listing 7). Rather than explicitly specifying which Traces should be plotted, the PlotSpec objects use tag-selection strings and directly query the SimulationResults object. TagViewer objects have a set of PlotSpecs that are used by default and automatically plot *Voltage*, *Current*, *Conductance* and other standard tags.

**Listing 6:**
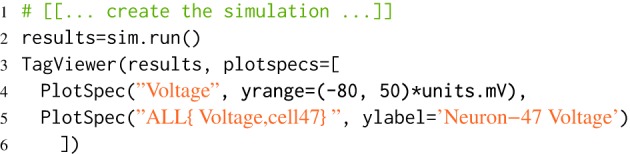
**Using TagViewer to define plots**. This listing will cause two axes to be displayed: the first will contain all Traces containing the tag *Voltage*, and the second will contain all Traces which have both *Voltage* and *cell47* as tags. PlotSpec objects can also be used to set other y-axis properties such as the range and label.

**Listing 7:**
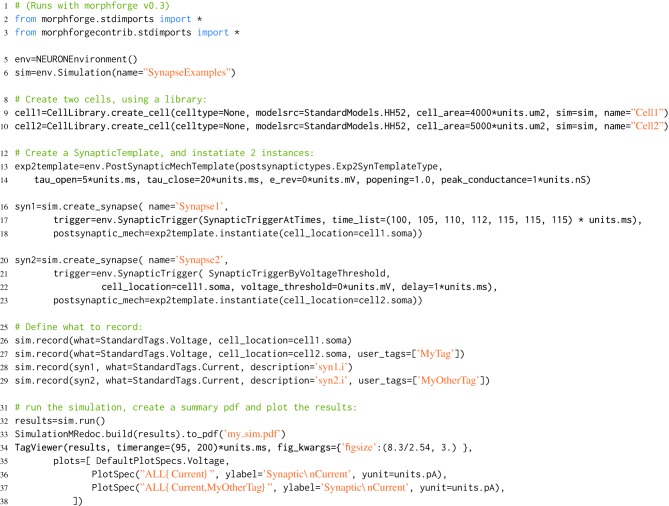
**An example of building a simple network simulation in morphforge**. Two Hodgkin-Huxley type neurons are created from a library (lines 9,10; described above). A template for a double-exponential post-synaptic receptor (PSR) is defined (lines 12-14). One instance of this PSR is created at the soma of *cell1*, and triggered by specific spike times (lines 16-18), and another is created at the soma of *cell2*, where it is triggered by an action potential in the soma of *cell1* (lines 20-23). The generated plots (lines 34-37) are shown in Figure 3, and the summary PDF file (line 33) is given in the supplementary material.

**Figure 3:**
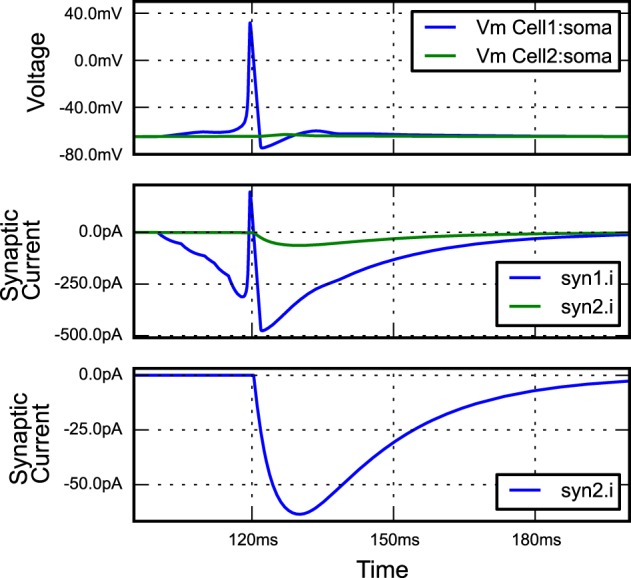
**The figure produced by the code in Listing 7**. The three plots correspond to the three PlotSpec objects defined in Listing 7, and pick out suitable records based on the *tag-selection strings* provided. (The legends in second and third plots were moved manually for clarity).

#### 3.2.7. Automatic model documentation

Models in computational neuroscience can involve complex equations, many units and large numbers of parameters; for example a Hodgkin-Huxley type sodium channel may involve 7 equations and 12 parameters. Often modeling involves adjusting parameters, which renders the tracking of model development difficult. Manually noting all the details of a complex simulation is unfeasible. One approach is to use version control, for example Sumatra (Davison, 2012). An alternative approach is to directly generate summaries of a simulation from the internal object-model to produce human readable output. The need for standard presentation formats for models has been recognized, even if exact formats have not yet been defined (Nordlie et al., 2009; Crook et al., 2012). morphforge can produce HTML and PDF document summaries from Simulation objects directly using the mredoc (Modular Reduced Documentation) library. This library is a high-level interface for producing documents containing images, tables, code snippets and equations for documenting mathematical models. Since simulations in morphforge can be populated with Synapse and Channel objects from different formats, which may be user defined, the summary architecture allows these objects to create summaries of themselves. The summary PDF file can contain summaries of individual neurons, (including 2D projections of morphologies and tables detailing parameters and channel distributions), and summaries of the dynamics of the channels and synapses (including tables of parameters and graphs of rate constants, steady-state values and time-constants). An example is given in Listing 6 and the resulting PDF file is given in the supplementary material.

### 3.3. Example: 2 neurons connected by a synapse

morphforge allows neurons to be connected by both gap junctions and chemical synapses. The numbers of synapses in a simulation can quickly become very large, and in order to allow efficient code to be generated, PostSynapticTemplate objects can be defined once and then instantiated between pairs of neurons, for example, as shown in Listing 7. morphforge also allows populations of neurons to be defined and connected together with synapses, using constructs similar to those used to connect single-compartment neurons in PyNN (not shown), although the object-model for this is experimental and likely to evolve. morphforge also supports spatial compartmentalization of multicompartmental neurons using a high-level notation (not shown). A fuller discussion of the software architecture with respect to synaptic models, populations and compartmentalization, as well as the design decisions taken for the implementation of the NEURON-backend can be found in the documentation and in the examples on the website.

### 3.4. Testing morphforge

The developmental approach to morphforge was a combination of defensive programming in conjunction with high-level functional testing (McConnell, 2004). For the functional testing, a new Simulator-TestData repository was created, which defines a set of scenarios in a simple, consistent, human and machine readable text file. Each scenario file describes the setup for a simulation, as shown in Listing 8. The specification allows parameters to be used in the description (for example <A>, <VS>, <C>, <GLK>, <EREV> and <I>), and defines what should be recorded (for example the voltage of cell1 as $V). The file defines which units to use for all the parameters and recorded values, and also defines the values that should be used for each parameter for parameter sweeps (not shown). The repository is designed for verifying the results from a simulator in two ways. Firstly, results can be compared against a set of hand-calculated results, which are given as a table in the scenario file (lines 11–20), and secondly, result traces from different simulators can be compared against each other.

**Listing 8:**
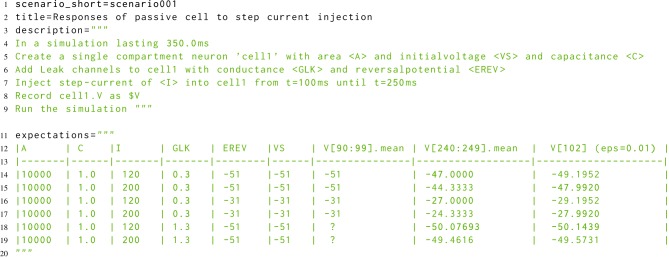
**A simplified example scenario from the Simulator-TestData repository, which defines the expected responses of a neuron with passive channels to a step current injection**. A human readable description of the simulation is given, as well as a table of expected values. The units of each parameter are given in a table in the file (not shown). Slice notation in the table columns represent times.

The scenario files were designed to be human readable, and it was found that by using techniques from Behavior Driven Development (BDD) (Chelimsky, 2010), it was possible to directly parse the description in the scenario files to build morphforge simulations. This removed the need to manually implement the tests in morphforge code and also raised the possibility of allowing neuroscientists to build complete simulations using a natural language syntax.

### 3.5. Building on the object model

**Figure 4:**
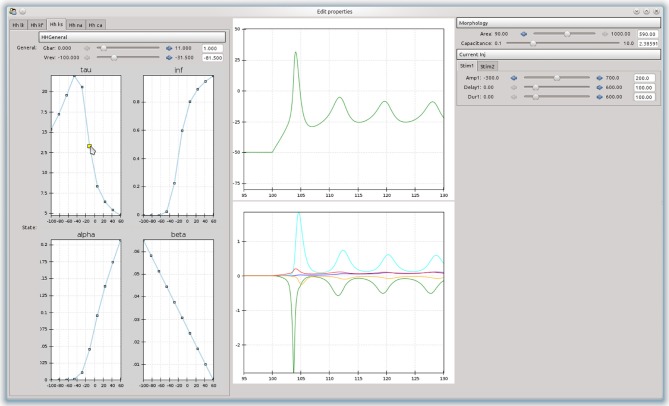
**A simple example of a graphical tool built on top of morphforge, which allows the effects of different parameters and rate-constant curves on the response of a neuron to step current injection to be interactively explored**.

An advantage of building an object-model over a stand-alone program is that it can be used as a basis to build more complex tools. For example, a simple tool was built that allowed the effects of channel parameters on the firing responses of a neurons in response to step current injections to be interactively explored (Figure 4). The curves are approximated as piecewise line segments, and the connecting points can be adjusted by dragging with the mouse. This was implemented as a custom Channel type for the NEURON backend. The graphs are interconnected and moving a point on either the time constant or steady state activation graphs will automatically adjust the forward and backward rate equation graphs. Changing any parameters in the simulation will cause the simulation to be run in NEURON and the results to be displayed in the center panel. The tool was built using the chaco^3^ library.

## 4. Discussion

morphforge was initially developed in order to allow computational models of small regions of the tadpole nervous systems to be easily built and reused and allow a large number of *in silico* experiments to be performed and maintained. It was generalized to an object-model for modeling and simulating small networks of multicompartmental neurons. The toolbox has a conceptually simple user-interface and also exposes a Python object model for further development. Since modelers at the cutting edge of science will often want to go beyond what was initially envisaged by a software package, morphforge does not try to define everything itself and instead allows users to provide custom functionality by writing specific plugins. morphforge is not a final polished piece of software, development is ongoing, and examples of simulations are provided. During its development, some decisions were taken because they were simple to implement, rather than being the best solutions and improvements are possible. (For example, if 500 Traces are recorded during a simulation, then 500 identical arrays will be created containing the time points, whereas a more efficient implementation would share these arrays between Traces.) morphforge provides a platform of loosely coupled tools for building and analysing simulations which allow complete model networks to be defined within a single Python file, without the need for external dependencies, whilst also allowing basic templates for components to be defined and variations reused across multiple simulation experiments.

Two routes exist for improving interoperability with existing software in the modeling ecosystem. The first would be to integrate with PyNN (Davison et al., 2008), to provide a single interface for multiscale modeling. This would allow a range of models from small networks of detailed multicompartmental neurons through to much larger networks of single compartment neurons to be implemented entirely within Python. The second is the representation of output data. In morphforge, the results from simulations are currently represented as Trace objects. Recently, the NEO library has defined a set of data structures for representing electrophysiological data, which allows files from a different vendors to be treated similarly^4^. Migrating morphforge to use these more standard data structures would allow the results from simulations to use the analysis routines for electrophysiological data more transparently.

Across all levels of nervous system research, from the tadpole to human brain, in order to build more complex models and validate them against experimental results, the infrastructure needed to manage data and simulations will become more complex. The scientific community has limited resources and we need to move toward standardized, flexible platforms that everyone can use and contribute to Crook et al. (2012). In biophysical modeling, the problems faced are not from understanding the concepts behind mathematical models but from the difficulties in quickly and reliably converting our ideas into simulations and then managing and communicating them (Wilson, 2006). To do this, we need to eliminate unnecessary complexity, avoid sources of trivial errors and provide libraries that allow us to succinctly build models without needing to regularly reinvent the wheel. We should develop and standardize tools and object-models that simplify mundane tasks to allow us to focus on the exciting scientific questions.

### 4.1. Data sharing

morphforge, including further examples and documentation is available at: https://morphforge.readthedocs.org. The Simulator-TestData repository is available from https://github.com/mikehulluk/simulator-test-data. morphforge uses Python 2.7 and runs on Linux.

### Conflict of interest statement

The authors declare that the research was conducted in the absence of any commercial or financial relationships that could be construed as a potential conflict of interest.

## Footnotes

^1^http://www.numpy.org.

^2^http://packages.python.org/quantities.

^3^http://code.enthought.com/projects/chaco/.

^4^http://neuralensemble.org/neo/.
