# Supplementary material for: morphforge: a toolbox for simulating small networks of biologically detailed neurons in Python
Source: Front Neuroinform. 2014 Jan 28;7:47. doi: 10.3389/fninf.2013.00047 (PMC3904074; doi:10.3389/fninf.2013.00047)
Supplement: Supplementary file 1 [file DataSheet1.PDF]

Simulation Summary

1 Simulation Summary: multicell_simulation010.py: SynapseExamples

Contents

1 Simulation Summary: multicell_simulation010.py: SynapseExamples **2**
- 1.1 Simulation Overview 4
  - 1.1.1 Diagram Overview 4
  - 1.1.2 Individual Cells 4
  - 1.1.3 Individual Synapses 4
- 1.2 Simulation Details 5
  - 1.2.1 Single Cell Details 5
    - Neuron:Cell1 5
    - Neuron:Cell2 7
  - 1.2.2 Channels Details 9
    - Summary of KChl (StdChlAlphaBeta) 9
    - Summary of LkChl (StdChlAlphaBeta) 11
    - Summary of NaChl (StdChlAlphaBeta) 11
  - 1.2.3 Synaptic Template Details 14
    - PostSynapticMech_Exp2Syn 14

2 Results **15**

List of Tables

- 1.3 Cell1:Morphology (Sections) 5
- 1.5 Cell1:Morphology (Regions) 5
- 1.7 Cell1:Passive Properties 5
- 1.9 Cell1:Channels 6
- 1.11 Cell2:Morphology (Sections) 7
- 1.13 Cell2:Morphology (Regions) 7
- 1.15 Cell2:Passive Properties 7
- 1.17 Cell2:Channels 8

1.1 Simulation Overview

1.1.1 Diagram Overview

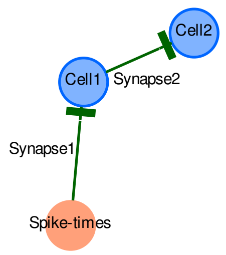

Figure 1.1

1.1.2 Individual Cells

Name	SA(um2)	#sections(#segs)	Regions(SA(um2):nseg)	#Pre/post-synapse	#Gap-juncs	Chls
Cell1	4000	1:8	soma(4000:8)	1 1	0	KChl NaChl LkChl
Cell2	5000	1:8	soma(5000:8)	0 1	0	KChl NaChl LkChl

1.1.3 Individual Synapses

Name	Trigger	PostSynaptic Cell	Receptor
Synapse1	At times: [100. 105. 110. 112. 115. 115. 115.] (ms)	Cell1@soma	Exp2Syn (onto Cell1@soma)
Synapse2	Cell1@soma: [threshold: 0.0 mV]	Cell2@soma	Exp2Syn (onto Cell2@soma)

1.2 Simulation Details

1.2.1 Single Cell Details

Neuron:Cell1

Table 1.3 – Cell1:Morphology (Sections)

ID	Tags	Lateral Surface Area (um2)	Region	nseg	L	diam (prox/dist)
0	soma	4000	soma	8	35.6824823231	35.7/35.7

Table 1.5 – Cell1:Morphology (Regions)

Region	Surface Area	#Sections
soma	4000.0	1

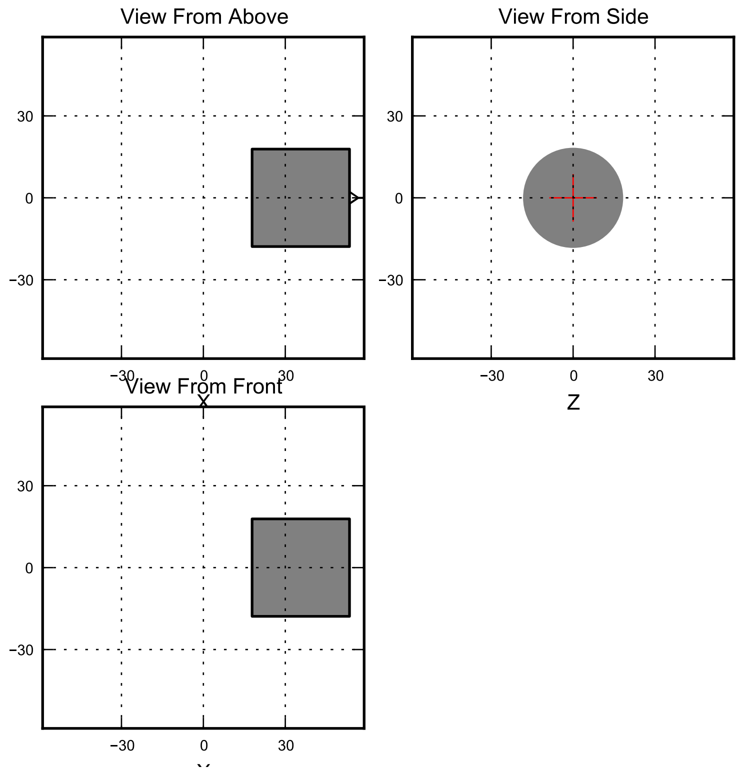

Figure 1.2

Table 1.7 – Cell1:Passive Properties

PassiveProp	Priority	Targetter	Value
AxialResistance	0	Default	80.0 ohmcm
SpecificCapacitance	0	Default	1.0 uF/cm2

Table 1.9 – Cell1:Channels

Mechanism	Priority	Targetter	Applicator
NaChl	10	Everywhere	Uniform Applicator:
KChl	10	Everywhere	Uniform Applicator:
LkChl	10	Everywhere	Uniform Applicator:

Neuron:Cell2

Table 1.11 – Cell2:Morphology (Sections)

ID	Tags	Lateral Surface Area (um2)	Region	nseg	L	diam (prox/dist)
0	soma	5000	soma	8	39.8942280401	39.9/39.9

Table 1.13 – Cell2:Morphology (Regions)

Region	Surface Area	#Sections
soma	5000.0	1

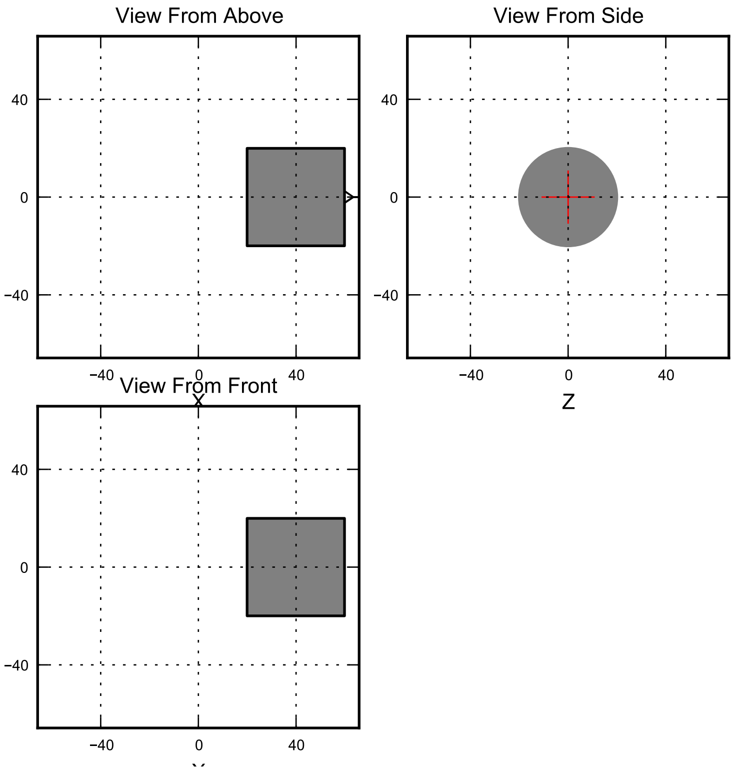

Figure 1.3

Table 1.15 – Cell2:Passive Properties

PassiveProp	Priority	Targetter	Value
AxialResistance	0	Default	80.0 ohmcm
SpecificCapacitance	0	Default	1.0 uF/cm2

Table 1.17 – Cell2:Channels

Mechanism	Priority	Targetter	Applicator
NaChl	10	Everywhere	Uniform Applicator:
KChl	10	Everywhere	Uniform Applicator:
LkChl	10	Everywhere	Uniform Applicator:

1.2.2 Channels Details

Summary of KChl (StdChlAlphaBeta)

$$g = gmax * n * n * n * n$$

$$i = g * (erev - V)$$

$$\frac{d}{dt}n = \frac{n_{\infty}(V) - n}{\tau_n(V)}$$

$$n_{\infty}(V) = \frac{\alpha_n(V)}{\alpha_n(V) + \beta_n(V)}$$

$$\tau_n(V) = \frac{1.0}{\alpha_n(V) + \beta_n(V)}$$

$$\alpha_n(V), \beta_n(V) = \frac{A + BV}{C + exp\left(\frac{D + V}{E}\right)}$$

Parameter	Value
Conductance (gmax)	36.0 mS/cm2
Reversal Potential	-77.0 mV

n	A	B	C	D	E
Alpha	-0.55	-0.01	-1.0	55.0	-10.0
Beta	0.125	0	0	65	80

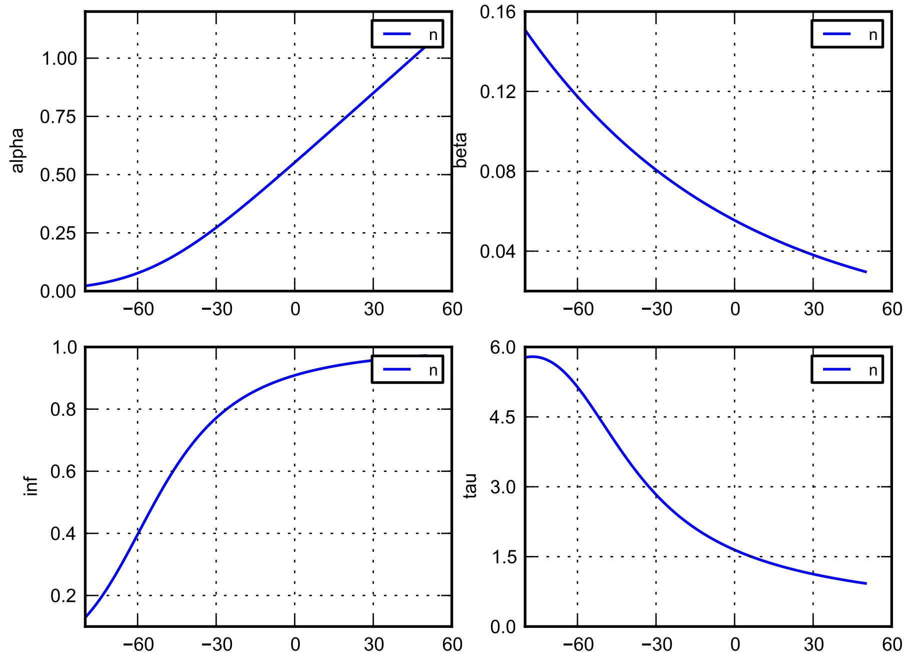

Figure 1.4 – The rate constants and resulting steady-state activation and time constants for n

Summary of LkChl (StdChlAlphaBeta)

$$g = gmax$$

$$i = g * (erev - V)$$

Parameter	Value
Conductance (gmax)	0.3 mS/cm2
Reversal Potential	-54.3 mV

Summary of NaChl (StdChlAlphaBeta)

$$g = gmax * m * m * m * h$$

$$i = g * (erev - V)$$

$$\frac{d}{dt}h = \frac{h_{\infty}\left(V\right) - h}{\tau_{h}\left(V\right)}$$

$$h_{\infty}\left(V\right) = \frac{\alpha_{h}\left(V\right)}{\alpha_{h}\left(V\right) + \beta_{h}\left(V\right)}$$

$$\tau_{h}\left(V\right) = \frac{1.0}{\alpha_{h}\left(V\right) + \beta_{h}\left(V\right)}$$

$$\alpha_{h}\left(V\right), \beta_{h}\left(V\right) = \frac{A + BV}{C + exp\left(\frac{D + V}{E}\right)}$$

$$\frac{d}{dt}m = \frac{m_{\infty}\left(V\right) - m}{\tau_{m}\left(V\right)}$$

$$m_{\infty}\left(V\right) = \frac{\alpha_{m}\left(V\right)}{\alpha_{m}\left(V\right) + \beta_{m}\left(V\right)}$$

$$\tau_{m}\left(V\right) = \frac{1.0}{\alpha_{m}\left(V\right) + \beta_{m}\left(V\right)}$$

$$\alpha_{m}\left(V\right), \beta_{m}\left(V\right) = \frac{A + BV}{C + exp\left(\frac{D + V}{E}\right)}$$

Parameter	Value
Conductance (gmax)	120.0 mS/cm2
Reversal Potential	50.0 mV

h	A	B	C	D	E
Alpha	0.07	0.0	0.0	65.0	20.0
Beta	1.0	0.0	1.0	35.0	-10.0

m	A	B	C	D	E
Alpha	-4.0	-0.1	-1.0	40.0	-10.0
Beta	4.0	0.0	0.0	65.0	18.0

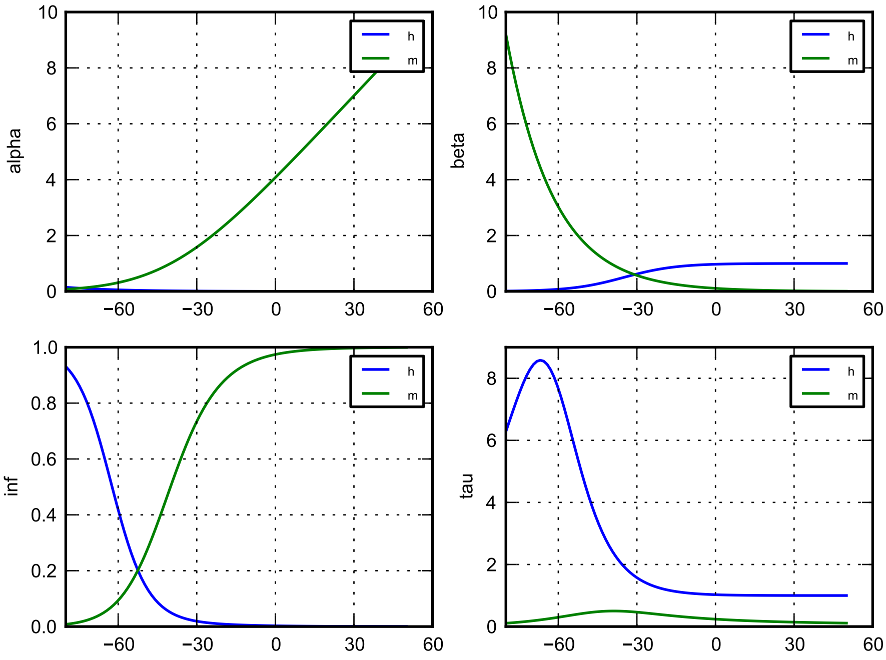

Figure 1.5 – The rate constants and resulting steady-state activation and time constants for h,m

1.2.3 Synaptic Template Details

PostSynapticMech_Exp2Syn

Needs double-checking!

Parameter	Value
e_rev	0.0 mV
peak_conductance	1.0 nS
popening	1.0
tau_close	20.0 ms
tau_open	5.0 ms

$$i = g_{peak} * (B - A) * \frac{1}{tc_{max}} * (V - E_{rev})$$

$$\frac{d}{dt}A = -A/tau_{open}$$

$$\frac{d}{dt}B = -B/tau_{close}$$

$$t_{max} = ln\left(\tau_{close}/\tau_{open}\right) * \left(\tau_{open} * \tau_{close}\right) / \left(\tau_{close} - \tau_{open}\right)$$

$$tc_{max} = exp\left(-t_{max}/tc_{c}lose\right) - exp\left(-t_{max}/tc_{o}pen\right)$$

2 Results

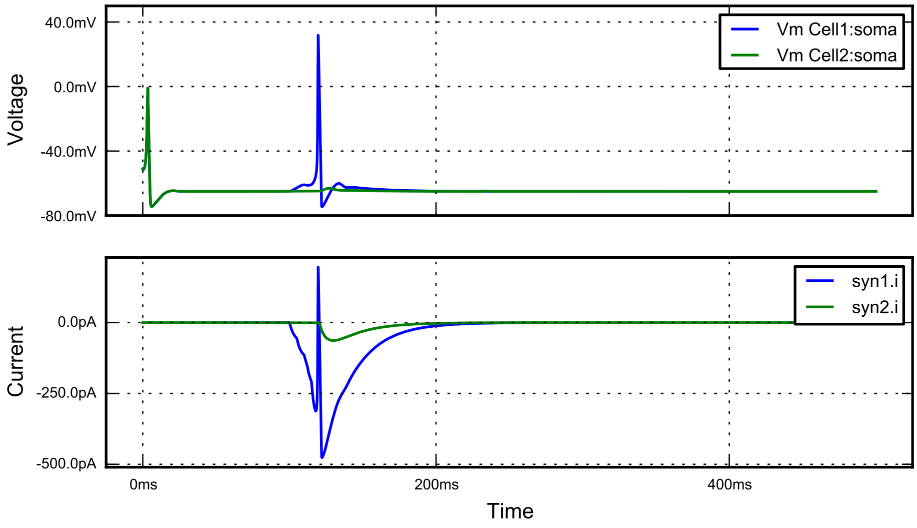

Figure 2.1
